# The Role of microRNA in Anaesthetics-induced Brain Injury: A Narrative Review

**DOI:** 10.4274/TJAR.2025.241739

**Published:** 2025-10-14

**Authors:** Elvan Öçmen, Bilge Karaçiçek, Burak İbrahim Arıöz, Hale Aksu, Şermin Genç

**Affiliations:** 1 Dokuz Eylül University Faculty of Medicine, Department of Anaesthesiology and Reanimation, İzmir, Türkiye; 2 Dokuz Eylül University, İzmir Biomedicine and Genome Center, İzmir International Biomedicine and Genome Institute, İzmir, Türkiye; 3 Dokuz Eylül University Health Sciences Institute, İzmir Biomedicine and Genome Center, Department of Neuroscience, İzmir, Türkiye

**Keywords:** Anaesthesia, anaesthetic agents, miRNA, neurotoxicity

## Abstract

Anaesthetics are commonly used agents during medical interventions and surgeries. Exposure to anaesthetic agents in late intrauterine life or early childhood may cause neurodegeneration in developing brains. Neuroapoptosis and neural inhibition provided by several mechanisms and microRNAs (miRNAs) have crucial roles in this milieu. miRNAs have critical roles in response to anaesthetic exposure. Through this review, we performed a systematic search of the PubMed database for studies on the role of anaesthetics in the brain and their relation with miRNAs. The terms “anesthetic”, “miRNA”, and “brain” were searched. Here we summarized the roles and interactions of miRNAs under exposure to anaesthetics *in vivo* and *in vitro* studies. Anaesthetic agents studied included sevoflurane, isoflurane, ketamine, and propofol. Many microRNAs were identified to have regulatory roles in anaesthesia-induced neurotoxicity. The literature study supports the idea that miRNAs play crucial functions in neuroprotection and neurotoxicity in anaesthesia administration. The exact role and implication of miRNA in anaesthesia neurotoxicity needs to be elucidated to gain more knowledge about the area. Several gaps in knowledge should be filled by conducting basic, clinical, and translational analyses in the future to decipher the definite role of miRNAs and their functions in the context of anaesthesia-induced neurotoxicity.

**Main Points**

• Anaesthetics cause microRNA (miRNA) expression changes in the brain.

• The exact mechanism of anaesthesia-induced neurotoxicity is still not certain. This review summarizes the miRNA expression changes that can be related to anaesthesia-induced neurotoxicity.

• miRNAs are not only related to neurotoxicity. Their expression changes can also result in the neuroprotection, addictive, and antidepressant properties of anaesthetics.

• It is too early to say which miRNA changes its expression after anaesthesia. But further studies and analysis may highlight one or a couple miRNAs related to anaesthesia-induced neurotoxicity. These could be the therapeutic candidates for neuroprotection.

## Introduction

Globally, over 300 million surgical operations and diagnostic or interventional tests that require anaesthesia or sedation occur annually. Recent evidence suggests that anaesthetics may cause neurodegeneration in the brain. In this review, our primary goal was to search for articles related to the role of microRNAs (miRNAs) in anaesthesia-induced neurotoxicity (AIN) and our secondary goal was to summarize articles about anaesthesia and miRNA changes in the brain.

**Figure 1:**
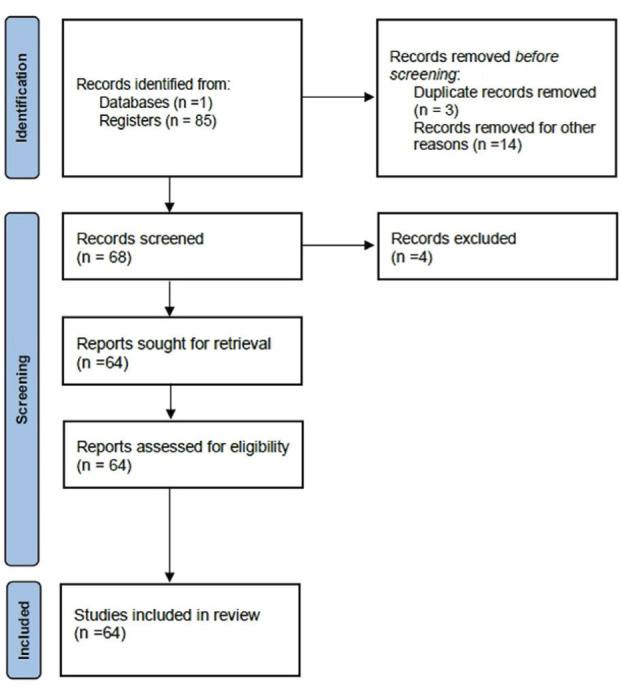
Study selection

In this study, we searched the PubMed database for studies on the role of anaesthetics in the brain and their relationship to miRNAs. The search was conducted in April 2022. The search terms were “anesthetic and miRNA and brain”, “miRNA”, and “brain”. Articles were screened by the authors to exclude duplicates and those that did not meet the inclusion criteria. The inclusion criteria was the articles about anaesthetics and miRNAs in the brain. The exclusion criteria were abstracts, conference lectures, case reports, reviews, biographies, and editorials. In addition, articles not published in English, those for which full texts were not available, and those not related to the brain were excluded. Data regarding the miRNAs involved, their expression status, and their roles in AIN were retrieved (Figure 1). This study aimed to identify miRNAs and their pathways associated with AIN. This review highlights possible mechanisms and miRNAs involved in AIN. This will help researchers to design future studies on this topic and to study possible protective agents.

### miRNA

miRNAs are small RNA molecules with a length of 19-25 nucleotides. Their main function is to regulate gene expression post-transcriptionally, and one miRNA can interact with hundreds of mRNAs.^1^ The first miRNA was discovered in 1993 by Lee et al.^2^ It has been shown that a smaller RNA product may bind to several sites of the 3′ untranslated region (UTR) of LIN-14 mRNA and mitigate LIN-14 protein expression without affecting its mRNA levels.^2^

### miRNA Biogenesis

Canonical miRNA biogenesis begins with the transcription of miRNA genes into primary miRNAs (pri-miRNAs) by RNA polymerase II. Then, pri-miRNA is cleaved into a precursor RNA, 70-120 nucleotides in length, by microprocessor, a multiprotein complex containing the ribonuclease enzyme Drosha and the RNA-binding protein DiGeorge syndrome critical region 8 (DGCR8). Newly produced pre-miRNAs are transported into the cytoplasm by exportin 5, which is a Ran-dependent nuclear transport receptor protein. Pre-miRNAs mature into 19-25 nucleotide-long duplex miRNAs by removing the terminal loop with Dicer-1, an RNase III enzyme. Duplexes are then separated, and mature miRNAs may interact with Argonaute proteins to form an RNA-inducing silencing complex, which can bind to the 3´ UTR of mRNAs and exert a silencing effect.^1^

### Role of miRNAs in Brain

miRNAs are found in the human brain and have distinct functions. They contribute to development, physiological functions, and human cognition by regulating transcription in specific regions of the brain.^3^ miR-124, one of the most abundant miRNAs in the brain, affects neural lineage differentiation by downregulating multiple mRNAs. Several studies have demonstrated that miRNAs show expression changes in many neurological diseases such as autism spectrum disorders, schizophrenia, frontotemporal dementia, Alzheimer’s disease, Parkinson’s disease, and neuropsychiatric diseases.^3^ Discovering miRNA-mRNA interactions in the brain may shed light on drugs that affect the central nervous system, such as anaesthetics.

### Sevoflurane

Sevoflurane is a commonly used inhalational anaesthetic, particularly during pediatric induction. Animal studies have widely reported sevoflurane-induced neuroapoptosis and cognitive dysfunction, especially during the brain growth spurt. The exact mechanism of sevoflurane-induced neurotoxicity (SIN) is unclear. In recent years, the roles of miRNAs and long non-coding RNAs (lncRNAs) in SIN have been studied. The hippocampus is the most studied part of the brain because it is closely associated with SIN.

### Sevoflurane *in vitro *Studies

To investigate SIN and the role of miRNAs, E14 mESCs, primary neuron culture, SH-SY5Y neuroblastoma cells, NSC culture from neonatal rat hippocampus, and HN-h, HCN-2, and HEK293 cell lines were used. In these studies, let-7a, miR-27a-3p, miR-188-3p, miR-183, hsa-miR-302e, miR-34a, miR-19-3p, miR-101a-3p, miR-133a, miR-410-3p, miR-214, and miR-128-3p expression were investigated. Except for miR-410-3p, the expression of all miRNAs studied *in vitro* increased after sevoflurane exposure. Changes in miRNA expression inhibited self-renewal, proliferation, and differentiation and they induced apoptosis and neuroinflammation. *Lin-28, PPAR-Ȣ, MDM2, Sox2, BDNF, OXR1, Wnt1, CCNA-2, CXCR5, Sox1, Peg13*, and *Sox13* were the target genes of miRNAs *in vitro* studies.^4, 5, 6, 7, 8, 9, 10, 11, 12, 13, 14^ A decrease in the expression of lncRNAs Gm15621 and Rik-203 was found to be related to SIN.^11, 15^

### Sevoflurane *in vivo *Studies

Eight microarray studies investigated changes in miRNA expression after sevoflurane anaesthesia. Five of these studies used rats. Of these, three focused on the hazardous effects of sevoflurane on children. therefore, they involved 7-day-old (P7) rats in the study, while two studies included adult rats. Two studies included P7 mice, and the newest microarray study chose P5-6 Rhesus monkeys so the Rhesus monkeys can reflect the miRNA changes in the mammalian brain. Although the concentration of sevoflurane was similar between studies (1.9-2.5%), the duration of anaesthesia varied between two and eight hours. The exact duration of anaesthesia for SIN is not clear, but a longer anaesthesia time could be closely related to neurotoxicity. Therefore, the duration of anaesthesia may be associated with changes in the expression of different miRNAs. Brain sections that have been investigated for miRNA expression are essential for understanding miRNAs' role in brain function. Six of the eight microarray studies used the hippocampus, which is closely related to learning and memory disturbances, for miRNA expression. One study selected the left cortex, while another investigated the frontal cortex. The timing of analysis after anaesthesia can result in the detection of different miRNA expression changes. Lin et al.^16^ showed this in their study. They found that the expression changes of only three miRNAs in the whole brain and four miRNAs in the hippocampus persisted for 2-4 months after sevoflurane anaesthesia. The exact time interval for the transient changes in miRNAs is unknown; therefore, after a few hours, some of the miRNA expression may return to normal. In addition to these methodological differences in microarray studies, changes in miR-125b-5p expression were observed in four studies.^17, 18, 19, 20^ Also, differential expression of miR-129-3p^16,17 ^and miR-448^18, 21^ has been detected in more than one study after sevoflurane anaesthesia.

Other studies on this topic chose miRNAs that are known to be related to brain function or neurodevelopment and analyzed their expression. Studies have shown that sevoflurane exerts differential effects on miRNAs. However, it can increase the expression of some miRNAs and decrease the expression of others. In a previous study, lncRNA Rik-203’s target miR-4661-3p was investigated in P6 mice, but the researchers could find no change in the expression of miRNA after sevoflurane anaesthesia.^11^ In contrast, Su et al.^12^ showed that sevoflurane could decrease miR-410-3p expression and cause apoptosis. Another study chose the target miR-133a of lncRNA Gm15621 and found that its expression increased with increasing concentrations of sevoflurane.^15^ LncRNA Rik-203 and its target miR-101a-3p were investigated. After sevoflurane anaesthesia, expression of miR-101a-3p was increased, while the expression of Rik-203 decreased.^11^ Zhao et al.^10^ investigated the miRNAs that bind to Cyclin A2 protein, which is downregulated after sevoflurane anaesthesia, showing that miR-19-3p was increased. Zhao et al.^9^ investigated the role of miR-34a in cortical development and found that inhibition of the miRNA decreases SIN. Another study showed that an increase in miR-96 expression is related to apoptosis after sevoflurane anaesthesia.^22^ A high concentration of sevoflurane (4.8%) was found to be related to increased expression of miR-183, resulting in SIN.^6^ Wang et al.^6^ reported that increased expression of miR-188-3p in the hippocampus is related to sevoflurane-induced apoptosis. Likewise, increased expression of miR-27a-3p^5^ and miR-27b^23^ induces SIN via different pathways. In addition, a recent study showed that increased expression of miR-214^13^ plays a role in SIN. Unlike these studies, Shan et al.^24^ showed that decreased expression of miR-30a, miR-31, miR-190a, and miR-190b is related to SIN in old rats.

### SIN Related Pathways

Many pathways have been implicated in SIN; therefore, different pathways related to miRNAs have been investigated. One of the most investigated genes is brain-derived neurotrophic factor (BDNF), which plays an important role in neural differentiation.^7, 10, 11, 13, 18^

Another commonly studied gene family is the Sox family, which plays a crucial role in cell renewal and differentiation.^7, 11, 25^ Neuroinflammation is one of the attributed reasons for SIN. miR-410-3p,^12^ miR-101a-3p,^11^ and miR-27a-3p^5^ are the miRNAs found to be related to SIN through neuroinflammation. Other studied pathways include the WNT signaling pathway,^9, 26^ phosphatidylinositol 3-kinase (PI3K) and protein kinase B signaling pathway,^12^ insulin-like growth factor 1,^22^ KEGG pathway,^16^ MDM2-p53 pathway,^6^ and LIMK1.^23^

### SIN Related Learning Memory and Function

Learning and memory tests are ways to observe the SIN’s clinical expression and evaluate cognition. The Morris Water Maze (MWM) is the most common learning and memory test, in which an experimental animal learns to locate the swimming platform in a tank filled with water. Sun et al.^26^ used MWM and showed that the WNT signaling pathway is the most strongly associated pathway to sevoflurane-induced cognitive dysfunction. Another study found a relationship between miR-410-3p and decreased latency time, and increased platform crossing in MWM after sevoflurane anaesthesia.^12^ Similar to these studies, the link between miRNAs and sevoflurane-induced cognitive dysfunction has been investigated using MWM.^5, 6, 9, 10, 22, 23^ Zhao et al.^10^ showed that the upregulation of miR‐19‐3p is associated with sevoflurane-induced learning and memory deficit, using the plus-maze discriminative avoidance task to demonstrate this association. Fujimoto et al.^18^ reported that sevoflurane anaesthesia could cause subsequent behavioral disorders at open field tests and contextual and cued fear conditioning tests by increasing rno-miR-632.

### SIN Related Concentrations in Neurotoxicity

The sevoflurane concentration used in these experiments is usually 1 MAC (2-3%), and the duration of anaesthesia varies between one-six hours.^12,13,16, 17, 18, 19, 20, 21,24,26^ These studies proved that even a relatively short duration of sevoflurane anaesthesia can cause SIN in an experimental setting. Studies that used repeated doses of sevoflurane anaesthesia (1 MAC for 2 hours over 3 consecutive days) also showed changes in the expression of miRNAs in the brain, resulting in SIN.^5, 9, 11, 25, 27^ Only one study failed to show miRNA expression change with 0.5 and 1 MAC sevoflurane, while 4.8% sevoflurane increased miR-183 expression.^6^

### miRNAs in Neuroprotective Role of Sevoflurane

In addition to its neurotoxic effects, sevoflurane exhibits neuroprotective properties under certain conditions. Shi et al.^28^ showed that sevoflurane preconditioning decreases the injury of cerebral ischemia by attenuating the upregulation of miR-15b. Similarly, Zhong et al*.*^29^ and Ding et al.^30^ reported the protective effect of sevoflurane on cerebral ischemia by altering the expression of miR-203 and miRNA-490-5p, respectively. Studies in glioma cell cultures have found that sevoflurane can suppress the proliferation, migration, and metastasis of glioma through miR-637,^31^ miR-124-3p,^32^ miR-146b-5p,^33^ miR-761,^34^ miR-34a-5p,^35^ and miR-7.^36^

### Isoflurane

Isoflurane is an inhalational anaesthetic, many experimental studies demonstrated that it could cause apoptosis, neurotoxicity, and learning-memory deficits. Rapidly proliferating and aged neurons are particularly vulnerable to the neurotoxic effects of isoflurane. Since the hippocampus is closely related to learning and memory, it is one of the most studied brain regions.

### Isoflurane *in vitro *Studies

Zhang et al.^37^ showed that isoflurane treatment represses the self-renewal of E14 embryonic stem cells through the mechanism involving miR-9’s direct target, E-cadherin. A study using hippocampal cell culture found that miR-448 increased after isoflurane anaesthesia, resulting in an increase in Bcl-x, a decrease in caspase-3, which causes neuronal apoptosis.^38^ Yang et al.^39^ reported that isoflurane can cause neuroapoptosis by decreasing miR-124 and BDNF in primary hippocampal neuron culture. Similarly, the increase in miR-142-5p expression after isoflurane was related to the inhibition of cell viability.^40^ The PTEN/PI3K/Akt pathway was investigated in a human neuroblastoma cell line, and the authors found that the decrease in miR-214 resulted in neuroapoptosis through this pathway.^41^

### Isoflurane *in vivo *Studies

In a miRNA microarray study, the hippocampus, was investigated after isoflurane anaesthesia. The authors found that 21 miRNAs increased in expression and 17 miRNAs decreased. They also showed that four miRNAs (miR-146a, miR-9, miR-143, and let-7d) are associated with learning memory deficits in the MWM. Additionally, they reported a relationship between downregulation of let-7d and increased amyloid-β expression.^42^ Shan et al.^24^ showed that a decrease in miR-30a, miR-31, miR-190a, and miR-190b expression in the hippocampus and mPFC is related to neuroapoptosis and impaired recognition and spatial memory. Similar to these two studies, it was shown that an increase in miR-448 expression in aged rats was related to learning and memory deficits and neurotoxicity.^38^

A study found that isoflurane decreased miR-124 expression in the hippocampus, increased EGR1, and caused learning-memory deficits and neuroapoptosis by increasing pro-apoptotic factors (cleaved-caspase-3 and Bax) and decreasing the anti-apoptotic factor Bcl-2 in neonatal rats.^39^ Two other studies found an increase in miR-142-5p^40^ and miR-191^43^ expression in the hippocampus. Li et al.^43^ showed that BDNF was the target gene of miR-191, whose increased expression results in neurotoxicity and learning-memory deficits.

Li et al.^44^ showed that overexpression of miR-24 decreases oxidative stress-related molecules such as superoxide dismutase, glutathione peroxidase and catalase, and neuroapoptosis *in vivo* and *in vitro*.

Whitaker et al.^45^ investigated the miRNA expression changes in female and male piglets’ hippocampi after isoflurane anaesthesia. Although they did not find a relationship between miRNA levels and isoflurane-induced neurotoxicity, they reported that there was no significant difference in miRNA expression between sexes. In addition to other isoflurane-related neurotoxicity studies, Fan et al.^46^ studied diabetic rats and found that miR-140-5p expression increased and caused neuroapoptosis through its target SNX12. In a study investigating the anaesthetic mechanism of isoflurane, the authors showed that among miRNAs, hsa-miR-17-5p had the highest expression level after isoflurane anaesthesia in the cortex. When they investigated sensory perception-related genes, they found that hsa-miR-16-5p, hsa-miR-424-5p, and hsa-miR-497-5p were the miRNAs with the highest expression changes after isoflurane exposure. The authors concluded that the mechanism of isoflurane anaesthesia could be related to the expression changes in miRNAs and related genes.^47^

### Ketamine

Ketamine is commonly used in pediatric surgery. In long-term exposure, ketamine causes hippocampal apoptosis and damages learning memory functions.^48^ Many studies have been conducted to understand the role of ketamine administration in neurotoxicity and miRNA interactions.

### Ketamine *in vitro *Studies

The functions of miR-107, miR-375, and miR-206 were investigated *in vitro*. Ketamine upregulates the aforementioned miRNAs, resulting in apoptosis, neurite degeneration, and neural death. Subsequently, the targeted miRNAs were downregulated via a lentiviral system, and the downregulation of miRNAs protected against ketamine-induced neural cell death and neural toxicity. In addition, BDNF was found to be a target gene for miRNAs.^48, 49, 50, 51^ In addition, ketamine downregulated the expression of miR-221-3p and attenuated IFN-α-stimulated NF-κB activation in human astrocytes (HA1800 cells).^52^

### Ketamine *in vivo* Studies

In miRNA microarray studies, the expression profiles of the hippocampus and prefrontal cortex, have attracted interest under *in vivo* conditions. miR-331-5p, miR-496-5p, miR-206, miR-98-5p, miR-148a-3p, miR-128-3p, miR-448-3p, miR-764-5p, miR-1264-3p, miR-1298-5p, and miR-1912-3p were differentially expressed in the miRNA microarray profiles.^53, 54^

Memory performance and cognitive function tests were performed for *in vivo *cognitive examination to understand the role of miRNAs in ketamine-induced neurotoxicity in the hippocampus. miRNAs overexpressed under ketamine exposure, which cause neuronal injury, were downregulated, or silenced to reverse neuronal injury. Inhibition of miR-124 enhances memory impairment in mice. In adolescent rats, the downregulation of miR-214 results in learning memory impairment. Downregulation of miR-34c improves memory in mice. Downregulation of miR-137 causes significant memory impairment, whereas overexpression of miR-137 rescues memory loss. miR-34a inhibition leads to enhanced memory impairment.^49, 55, 56, 57, 58^

### The role of miRNA in Antidepresant Effect of Ketamine

Ketamine has a remarkable and persistent antidepressant effect . A study showed that miRNA-206 might be a therapeutic target for the antidepressant effects of ketamine.^59^ Another study found that ketamine resulted in increased expression of miR-98-5p, and downregulation of miR-98-5p resulted in a decrease in the antidepressant effect of ketamine in a mouse model.^60^ One study investigated the alteration of miR-29b-3p in rat brains. A significant increase in miR-29b-3p expression was observed in the prefrontal cortex of normal rats after ketamine exposure. Recombinant adeno-associated virus-mediated overexpression of miR-29b-3p resulted in recognizable relief of depressive behaviors in rats and lower expression of metabotropic glutamate receptor 4 (GRM4).^61^ After administration of an antidepressant dose of ketamine, the miRNAs 448-3p, 764-5p, 1264-3p, 1298-5p, and 1912-3p clusters were upregulated.^62^

### The role of miRNA in Ketamine Addiction

The hippocampus is a critical area in the brain involved in addiction. Li et al.^63^ showed hippocampal miR-331-5p was significantly decreased in the ketamine group, and upregulated in the ketamine+rhynchophylline group. In conclusion, miR-331-5p was found to be an important regulator of Nurr1, and rhynchophylline is involved in ketamine addiction, via the miR-331-5p/Nurr1/BDNF pathway or the inhibition of CREB phosphorylation.

### The role of BDNF Signaling in miRNA Alteration in the Brain by Ketamine

BDNF is a common target gene of miRNAs. Mir-107 inversely regulates BDNF. siRNA-mediated BDNF inhibition, reversed the protective effect of miR-107 downregulation on ketamine injury.^48^ Similarly, ketamine upregulated hsa-miR-375 and downregulated BDNF. BDNF is inversely regulated by ketamine-induced neural cell death and toxicity in hESC-derived neurons.^51^ miR-206 overexpression improved ketamine-induced upregulation of BDNF. This indicates that miRNA-206 may be a therapeutic target for the antidepressant effects of ketamine.^59^ miR-206 is the target for KCNQ1OT1 and downregulates its expression level, indirectly increasing the expression level of BDNF, and thus has a protective role in neural injury.^50^

### Propofol

Propofol (2,6-diisopropyl phenol) is a common sedative/anaesthetic.^64^ Propofol acts rapidly and has antiemetic properties.^65^ Aside from its sedative use, propofol is used in the management of refractory status epilepticus.^66^ Both advantageous and harmful effects of propofol via miRNAs have been reported in this context.

### Propofol *in vivo *Studies

An important miRNA that has caught the attention of researchers is miR-34a. Inhibition of miR-34a ameliorated propofol-induced impaired learning and memory function, as well as apoptosis, in Sprague-Dawley rats.^67^ In another study, it was demonstrated that propofol increased neuronal injury in the hippocampus of rats via miR-34a expression, and dexmedetomidine mitigated injury by targeting SIRT1 and PI3K/Akt signaling.^68^ Propofol injections in seven-day-old Sprague-Dawley rats resulted in miR-132 expression downregulation and a reduction in dendritic spine density in the hippocampus, and thus, impairment of learning and memory.^69^ Wang et al.^70^ verified that propofol caused elevated neurocyte damage by diminishing circRNA001372 levels while augmenting IL-1B, IL-6, IL-17, and IL-18. Subsequently, miR-148b-3p, an antagonist of circRNA001372, reversed the possible protective effects of circRNA001372. Propofol was shown to decrease inflammation through the miR-145-3p/NFATc2/NF-κB pathway.^71^

### Propofol *in vitro *Studies

Several *in vitro* studies have demonstrated a highly intertwined relationship between propofol and Rno-miR-665. After propofol treatment, Rno-miR-665 was upregulated in primary astrocyte cultures, and it represses BCL2L1; therefore, neuroprotection was decreased.^72, 73, 74, 75^ Li et al.,^67^ proved that propofol enhanced miR-34a levels with subsequent neurotoxicity, and inhibition of miR-34a rescued negative effects through the MAPK/ERK pathway. A study stated a protective role of propofol. It inhibited pro-inflammatory factors such as NO, TNF-α, and IL-6 via mitigating miR-155 expression, which targets SOCS1.^76^ In addition, propofol inhibited proinflammatory microglia activation via miR-221 and miR-222 downregulating IRF2 in BV2 microglia.^77^ miRNA profile screening of primary microglia suggested that propofol inhibited LPS-induced pro-inflammatory responses through the miR-106/Pi3k/Akt pathway.^78^ Propofol mitigated cerebral ischemia/reperfusion injury by downregulating MALAT1 and upregulating miR-182-5p.^79^ Moreover, propofol alleviates cerebral ischemia/reperfusion injury via the SNHG14/miR-30b-5p axis.^80^ Additionally, some screening studies show miRNA expression profiles, all miRNAs altered by propofol.^20, 21, 81^ In two separate studies, miR-455-3p decreased neurotoxicity induced by propofol via HOTAIR/NLRP1^82^ and EphA4.^83^ Propofol also blocked apoptosis in hippocampal neurons via the miR-15a-5p/NR2B/ERK1/2 axis.^84^

### Conclusion and Future Perspectives

**Figure 2:**
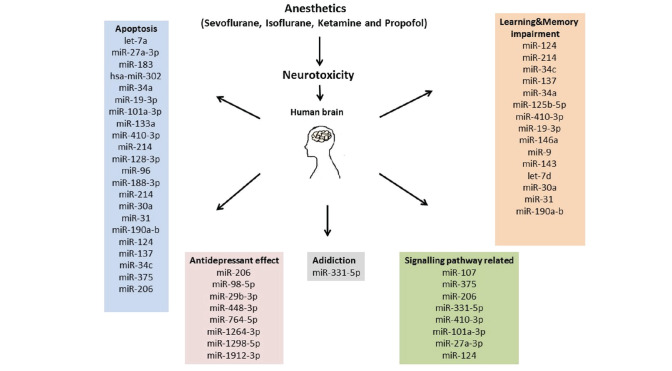
Summary of some miRNAs related to anaesthetic neurotoxicity

Every year, many adults and infants are exposed to anaesthetics because of their medical needs. Herein, we compile comprehensive data on the role of miRNA in anaesthetics’ effects (Figure 2). Animal studies demonstrated that anaesthesia induces neuroapoptosis and cognitive dysfunction during brain growth. According to studies on databases, sevoflurane, isoflurane, ketamine, and propofol are the most studied anaesthetic agents in relation to miRNA and neurotoxicity. In the light of the *in vivo* and *in vitro* studies, there is no doubt that substantial improvement has been made in understanding the role of miRNAs in anaesthesia neurotoxicity, but we are still in the early days, and so there is a lot of work to be done to fully resolve the neurobiology of miRNAs. Although we know that many miRNAs are related to neurotoxicity in the brain after anaesthesia exposure, the molecular mechanisms and degree of specific miRNAs involved in the onset and progression of these neurotoxicities are unknown.

A remarkable proportion of studies have stated that anaesthetics mostly affect the hippocampal region, where neurodegeneration and neuroapoptosis occur. Cognitive dysfunction and learning memory problems have also been observed.

Rodent studies have shown that sevoflurane anaesthesia can induce neurotoxicity and learning and memory deficits. The results of preclinical studies cannot be applied to clinical practice directly. The duration of anaesthesia, repeated exposure, the dose of the anaesthetic, the use of multiple agents, and the age of the animal are factors influencing cognitive dysfunction in preclinical studies. The results of clinical studies are contradictory.

The altered miRNA expression in the brain after anaesthesia could be an underlying mechanism in the sevoflurane-induced cognitive dysfunction. The studies that investigated the effect of sevoflurane on cognitive dysfunction through miRNAs have varying results. Investigating the miRNA expression in response to sevoflurane anaesthesia has several challenges. First, different methods were used in different studies. Second, some studies investigated the whole miRNA expression in the hippocampus, while others investigated specific miRNA alterations. Third, the duration and concentration of sevoflurane anaesthesia were different in each study. Although additional consistency is needed for more meaningful results, these preclinical studies showed that sevoflurane-induced cognitive dysfunction is related to alterations in miRNA expression. Additionally, the use of miRNA-specific knockout animals in such studies should be considered. miRNAs are becoming biomarkers for the diagnosis and prognosis of other conditions; thus, they can be the targets of therapy for AIN and cognitive dysfunction.

Both *in vitro* and *in vivo* studies have investigated the effects of ketamine on neurotoxicity and miRNA interactions. *In vivo *studies have demonstrated that ketamine causes neurodegeneration in hippocampal neurons, which is followed by an increase in apoptosis and cognitive dysfunction.

One of the critical aspects of miRNAs is the diversity of technologies used for miRNA detection. In contrast to common methods like real-time fluorescent quantitative polymerase chain reaction (PCR) and digital PCR technology, *in situ *hybridization and new microarray methods have been successfully established and have started to be widely used. More recent advances for miRNA detection include electrochemical detection based on enzymatic signal amplification, rolling ring amplification, and nanoparticle technology.^85^

Due to scientific and technological achievements, some miRNAs can be used as candidate biomarkers, allowing earlier detection and possibly a higher rate of success for a neuroprotection strategy. Therefore, future work should focus on developing a non-invasive and effective method to deliver miRNA “drugs” into the injured brain to protect it from neurotoxicity.

A comprehensive functional characterization of miRNAs in the context of the interaction among themselves (miRNA/miRNA) with other non-coding RNA species, especially IncRNAs, has attracted attention. This is because carnici carried out an extensive analysis of the transcriptional landscape in mouse brains, and his colleagues demonstrated that a high number of lncRNAs are involved in the regulation of several target genes in the brain.^86^ Therefore, miRNA-lncRNA interactions and their effect on the target gene are important for miRNA research, making it necessary to elucidate the cause/consequence relationship of their regulation in anaesthesia-related neuroprotection and neurotoxicity.

In conclusion, while the literature supports that miRNAs play crucial roles in neuroprotection and neurotoxicity during anaesthesia administration, the exact role of miRNAs in anaesthesia neurotoxicity needs to be elucidated. Moreover, whether a single miRNA or a combination of miRNAs should be considered the “ideal” therapeutic candidate for neuroprotection and treatment against neurotoxicity requires further studies and more in-depth analysis.
